# A Cohort study evaluation of maternal PCB exposure related to time to pregnancy in daughters

**DOI:** 10.1186/1476-069X-12-66

**Published:** 2013-08-20

**Authors:** Chris Gennings, Caroline Carrico, Pam Factor-Litvak, Nickilou Krigbaum, Piera M Cirillo, Barbara A Cohn

**Affiliations:** 1 Department of Biostatistics, School of Medicine, Virginia Commonwealth University, Richmond, VA, USA; 2 Department of Epidemiology, School of Public Health, Columbia University, New York, USA; 3 Child Health and Development Studies, Center for Research on Women’s and Children’s Health, Public Health Institute, Berkeley, CA, USA

**Keywords:** Endocrine disruptors, Polychlorinated biphenyls, Prenatal exposure

## Abstract

**Background:**

Polychlorinated biphenyls (PCBs) remain ubiquitous environmental contaminants. Developmental exposures are suspected to impact reproduction. Analysis of mixtures of PCBs may be problematic as components have a complex correlation structure, and along with limited sample sizes, standard regression strategies are problematic. We compared the results of a novel, empirical method to those based on categorization of PCB compounds by (1) hypothesized biological activity previously proposed and widely applied, and (2) degree of ortho- substitution (mono, di, tri), in a study of the relation of maternal serum PCBs and daughter’s time to pregnancy.

**Methods:**

We measured PCBs in maternal serum samples collected in the early postpartum in 289 daughters in the Child Health and Development Studies birth cohort. We queried time to pregnancy in these daughters 28–31 years later. We applied a novel weighted quantile sum approach to find the bad-actor compounds in the PCB mixture found in maternal serum. The approach includes empirical estimation of the weights through a bootstrap step which accounts for the variation in the estimated weights.

**Results:**

Bootstrap analyses indicated the dominant functionality groups associated with longer TTP were the dioxin-like, anti-estrogenic group (average weight, 22%) and PCBs not previously classified by biological activity (54%). In contrast, the unclassified PCBs were not important in the association with shorter TTP, where the anti-estrogenic groups and the PB-inducers group played a more important role (60% and 23%, respectively). The highly chlorinated PCBs (average weight, 89%) were mostly associated with longer TTP; in contrast, the degree of chlorination was less discriminating for shorter TTP. Finally, PCB 56 was associated with the strongest relationship with TTP with a weight of 47%.

**Conclusions:**

Our empirical approach found some associations previously identified by two classification schemes, but also identified other bad actors. This empirical method can generate hypotheses about mixture effects and mechanisms and overcomes some of the limitations of standard regression techniques.

## Background

Despite their ban in the 1970s, polychlorinated biphenyls (PCBs) remain ubiquitous environmental contaminants due to their chemical stability and lipophilic properties [1]. In fact, one or more PCBs are detectable in over 80% of blood samples in contemporary participants in the National Health and Nutrition Examination Survey (NHANES; e.g., [2] studying 2001–02 NHANES data for women of child bearing age). PCBs are hormonally active, and there is mounting evidence that *in utero* PCB exposure shortens length of gestation in humans [1] and is inversely associated with fetal growth [3]. Our focus herein from a prospective study, prenatal exposure to PCBs may disrupt pregnancy in humans [4].

Fecundability (probability of pregnancy in each cycle) has been used as a sensitive marker to identify reproductive hazards in humans [5]. Increased time to pregnancy (TTP) may indicate a problem at one or more of several stages of human reproduction, including gametogenesis, transport of gametes in both male and female reproductive tracts, fertilization, migration of the zygote to the uterus, implantation and early survival of the conceptus [4,5]. Increased TTP is most likely multifactorial with risk factors in utero as demonstrated by effects of prenatal diethylstilbestrol exposure [6], childhood and puberty as demonstrated by effects of treatment for childhood cancer [7], and adult life where smoking has been extensively investigated [8].

Cohn et al. [4] tested the hypothesis that *in utero* exposure to PCBs alters TTP in humans using data from the Child Health Development Studies (CHDS). Their data included measures of maternal exposures in blood samples collected near delivery (days 1–3 after daughter’s birth during 1960–63) in relation to her daughter’s TTP decades later. Their analysis was based on a previous categorization of PCB congeners by Wolff et al. [9] based on functionality and inclusion in CHDS maternal serum samples:

● Group 1: potentially estrogenic and persistent: PCB 101, 187, 201;

● Group 2: potentially anti-estrogenic, immunotoxic, dioxin-like

o Group 2A: non-ortho or mono-ortho in structure: PCB 66, 74, 105, 118, 156, 167

o Group 2B: di-ortho and limited dioxin-like activity: PCB 138, 170

● Group 3: phenobarbital, CYP1A and CYP2B inducers: PCB 99, 153, 180, 203, 183.

Serum concentrations for two uncategorized PCBs (PCB56 and PCB146) were also included in the analysis herein.

The model building strategy conducted by Cohn et al. [4] included two steps. Step 1 entered congeners in a single model with potentially opposing effects (e.g., estrogenic, Group1, and anti-estrogenic, Group 2) to avoid “overlooking any associations for estrogenic compounds that might be confounded by the presence of correlated anti-estrogenic compounds.” The second step included remaining congeners from Group 3. Non-significant congeners were removed from the model when their p values exceeded 0.10. The initial model was adjusted for whether the daughter was breastfed (yes or no) and race (African American vs all other); the final model was further adjusted for the following maternal variables: age, body mass index, and lipids (triglycerides, cholesterol) to determine whether these altered associations observed. Following this strategy, PCB congeners 187, 156, and 99 in mother’s serum were associated with longer TTP in their daughters; PCB congeners 105, 138 and 183 were associated with shorter TTP.

Using the same data, our objectives are to use a holistic empirical approach to investigate the association between PCB functionality and TTP in humans. Recognizing the complex correlation structure among the PCB congeners (Additional file 1: Table S1) and with a limited sample size, standard regression strategies are problematic (e.g., [10]). Instead, we use a nonlinear weighted quartile sum (WQS) approach [11] nested within a parametric proportional hazards model. The method is nonlinear due to the empirical estimation of a weighted sum of quartiles with the typical regression coefficients. The index is based on quartiles of mixture components to reduce the impact of extreme values in typically right-skewed concentration distributions. Bootstrap distributions of the estimated weights are used to identify the strength of association between the Wolff et al. categories and TTP, the degree of chlorine substitution and TTP, and to define an ‘average weighted quartile sum index’ that is associated with TTP. The weighted index stabilizes the ill conditioning in the analysis due to multicollinearity [11].

Here we contribute to the literature in two significant fashions. First, we consider maternal prenatal exposures and TTP in offspring from a well- documented birth cohort. Previous work (e.g. [12]) has considered exposures and TTP only in the parental generation. Second, we analyze PCBs as mixtures using a novel empirical method.

## Methods

### Description of study sample

As described in Cohn et al. [4], the study sample consists of women born between 1960 and 1963 in the Oakland, California area, whose mothers were enrolled in the Kaiser Permanente Health Plan. These women and their mothers were among participants in the Child Health and Development Studies (CHDS), an investigation of prenatal determinants of infant, child, adolescent and adult health. Subjects were respondents to a follow-up study conducted in 1990–1991 which investigated prenatal determinants of TTP. Women were ages 28–31 years (65% participation rate after 30 years since their birth) when they completed a questionnaire on the number of non-contracepting menstrual cycles that preceded their most recent pregnancy; or the number of non-contracepting cycles during which they were at risk for pregnancy even when pregnancy was not achieved. Women who did not plan their pregnancies as well as women who did plan their pregnancies were included in the study as long as they could report their TTP, reducing planning bias. Details of the study sample are given below.

CHDS daughters born during 1960–1963 who resided in the San Francisco Bay area through ages 15–17 (N = 1,003) were eligible for this study if their mothers had reported information on smoking during pregnancy (N = 991). TTP data was originally collected for a study of prenatal tobacco exposure conducted by Dr. Pam Schwingl for her dissertation research. Of these 991 eligible women, 345 did not participate for the following reasons: no address, N = 117, no response to mail, N = 197, deceased or institutionalized, N = 7, refused, N = 24. There were 646 participants in the study (65% of 991 eligible), of which 357 were excluded from analysis of TTP for the following reasons: respondent was never at risk for pregnancy (N = 163), respondent could not estimate TTP due to sporadic birth control use or birth control failure (N = 119), incomplete questionnaire (N = 47), insufficient prenatal serum for assays (N = 18), younger sister (N = 5), under age 16 at most recent pregnancy “attempt” (N = 5).

Women in the following categories were included in the present study whether or not they had planned their pregnancy, as long as they could report TTP: women who omitted birth control just once, women who became pregnant during an interval between birth control methods or after stopping birth control, women who became pregnant when breast feeding, women who never used birth control, women who were attempting pregnancy after a prior pregnancy, and women who never became pregnant, but had been at risk for pregnancy for a known interval, whether or not they desired to become pregnant. Women were asked to report their TTP for their most recent pregnancy in order to minimize recall errors. Among the 289 women included in this report, 43% of women reported TTP for their first pregnancy, 26% for their second pregnancy, 15% for their third pregnancy, 8% for their fourth pregnancy, and 6% for their fifth or more, with 2% where gravidity before attempt was unknown. Women reporting on time to first pregnancy include women who were at risk for pregnancy, but failed to conceive.

In human observational studies where being at risk for pregnancy is not an experimental condition, there are expected differences between women who can report TTP and women who cannot. The two primary groups of women who are deleted from the TTP analysis are women never at risk for pregnancy (N = 163) and women who became pregnant and could not answer about TTP (N = 119). Compared to the analysis sample, women never at risk for pregnancy had higher personal incomes, were much more likely to have never been married, and were more highly educated and more likely to be Asian. Compared to the analysis sample, women who used birth control sporadically and could not report their TTP were more likely to be African American or Asian, slightly more likely to be never married, somewhat better educated, but were similar to the analysis sample for personal income. These differences result from personal, social and economic factors related to reproductive behavior, and are not surprising. Our results apply to those women who were at risk for pregnancy and could provide a TTP, including women who planned their pregnancies and women who did not plan their pregnancies. This research was approved by the Institutional Review Board of the Public Health Institute, and study subjects gave informed consent.

### Exposure measurements

*In utero* exposure to PCBs among CHDS daughters was estimated by assays of serum samples drawn during the early postpartum period (days 1–3) from their mothers. PCBs were assayed in the laboratory of Dr. Mary Wolff. Details are provided in Cohn et al. [4].

### Statistical analysis

Time to pregnancy was modeled using a two-parameter Weibull baseline hazard model, allowing for the “hazard” of pregnancy to be monotonic (increasing, decreasing, or constant) in a Weibull proportional hazards model. The model was parameterized using a nonlinear weighted quartile score regression strategy (described below). Following Cohn et al. [4], the initial model included two covariates: race (African American vs all other) and whether the daughter was breastfed (yes or no). The final model was further adjusted for maternal variables: age, body mass index, and maternal lipids (triglycerides, cholesterol) to determine whether these altered associations observed. The assumption of a Weibull hazard model with proportionality across covariate categories was verified using diagnostic plots of log(−log(S(t)) vs log(t), where S(t) is the survival function.

Construction of weighted quartile score Weibull proportional hazards model:

A quartile score (robust to extreme values) was calculated for each chemical: values in the 1^st^ quartile, q = 0; values in the 2^nd^ quartile, q = 1; values in the 3^rd^ quartile, q = 2; values in the 4^th^ quartile, q = 3. The body burden from the set of PCBs was calculated by summing the quartile scores across chemicals. However, the potency of the chemicals vary substantially; the inclusion of less potent chemicals may add “noise” in the score and mask the signal from more potent chemicals, and this problem may be exacerbated when the less potent chemicals are more prevalent than more toxic chemicals. Therefore, we used weighted quartile score regression within a Weibull proportional hazards model. The general approach was characterized by Carrico [11]; who extended the work of Gennings et al. [13] and Christensen et al. [14] with the addition of a bootstrap step for determining the weights.

Define *t* as the time (i.e., cycle) of pregnancy or last cycle of not pregnant. For each daughter, data are available for serum PCB congener concentrations from her mother within 3 days of her delivery along with covariates. A nonlinear Weibull proportional hazards model was parameterized as:

(1) $$h\left(t;q,z\right)=\left\{\gamma \lambda t^{\gamma -1}\right\}\exp\;\left[\beta_{1}\;\left(\sum_{j}\;w_{j}q_{j}\right)+\sum_{k}\;\theta_{k}z_{k}\right]$$

where *t* is the cycle number; γ and λ are Weibull parameters for shape and scale, respectively; q_j_ is the quartile score for the j^th^ chemical; the *w*_*j*_ ∈ [0, 1] are estimated using a nonlinear programming optimization algorithm subject to the constraint that ∑ _*j*_*w*_*j*_ = 1. The z’s represent covariates (e.g., race, breast fed indicator) with the corresponding unknown parameters estimated using maximum likelihood estimation. Initial estimates for the covariates were based on estimates from a proportional hazards model without adjusting for PCBs (from Proc LifeReg in SAS version 9.2); the weights were initially set to be equal and constrained to sum to 1.0. The trust region method was used for the primary analysis as it is found to be stable for small to moderate sample sizes [15]. To ensure that the results were not dependent on the choice of the trust region method, the conjugate gradient method, the Newton–Raphson, the Nelder-Mead Simplex, and the Newton–Raphson with ridging as available in Proc NLP in SAS (version 9.2) were also assessed and the results were consistent using all algorithms. Following the approach of Carrico [11], the distributions of the weights were accommodated using 1000 bootstrap samples as follows:

● Bootstrap samples of size 289 (as in the observed data) were randomly generated by sampling with replacement and a set of weights and regression coefficients were estimated for each;

● In some datasets, without constraining the algebraic sign of *β*_1_, the slope estimate for the weighted PCB score was estimated to be <0; in other datasets, the slope estimate for another PCB score was estimated to be >0.

● Histograms for the weights were constructed for the first 1000 bootstrap analyses with positive and significant slope estimates (p < 0.05), and for the first 1000 bootstrap analyses with negative and significant slope estimates.

● Average weighted quartile sum indices were constructed based on the mean weights from 1000 bootstrap samples with positive and significant slope estimates and from 1000 bootstrap samples with negative and significant slope estimates. These indices were tested for significance in the original dataset.

The weights to be averaged were selected based on an indication of significance in the bootstrap sample such that the weights are interpretable; i.e., when the slope estimate is near zero, the weights are not informative. Conditional on the average bootstrap weights, when *β*_1_ in equation (1) is positive and significant, we claim the weighted quartile score is associated with an increase in the “hazard” of pregnancy, i.e., shorter TTP; when *β*_1_ is negative and significant, we claim the weighted quartile score is associated with a decrease in the hazard of pregnancy, i.e., longer TTP. Both a positive case and a negative case were estimated separately from the observed data. From the model in (1) the relative hazard associated with an increase of 1 unit of the PCB scores was exp(β_1_).

Sums of estimated average weights were tabulated within the Wolff et al. PCB categorization considered by Cohn et al. [4] and the degree of chlorination considered by Kezios et al. [1]. These sums may be interpreted as the strength (proportion) of the association due to the PCB category.

Ideally, when the sample size is large enough, the weighted quartile score regression strategy includes randomly splitting the data into a ‘test’ dataset and a ‘validation’ dataset. The test dataset is used to estimate the weights and the significance of slope parameters is based on the validation data where the weighted score is fixed. However, in the current case, the sample size is not considered large enough to (1) provide stable estimation of the weights in the test dataset; and (2) maintain adequate power in the validation dataset to test for the significance of *β*_1_.

## Results

Of the 289 women included in the study sample, 239 women reported the number of non-contracepting cycles resulting in pregnancy. Fifty women reported either 12 or 13 non-contracepting cycles from which pregnancy was not achieved – a 17% censoring rate. The estimated median time to pregnancy was 2 cycles with the middle 50% (25^th^ percentile to 75^th^ percentile) between 1 and 6 cycles. The young age of the respondents (ages 27–31 at interview) resulted in short time interval between time of interview and their most recent pregnancy, or most recent at risk period (median interval of 3 years, with 25% reporting on a pregnancy or attempt time of 1 year of less, and 75% within the last 5 years.

**Figure 1:**
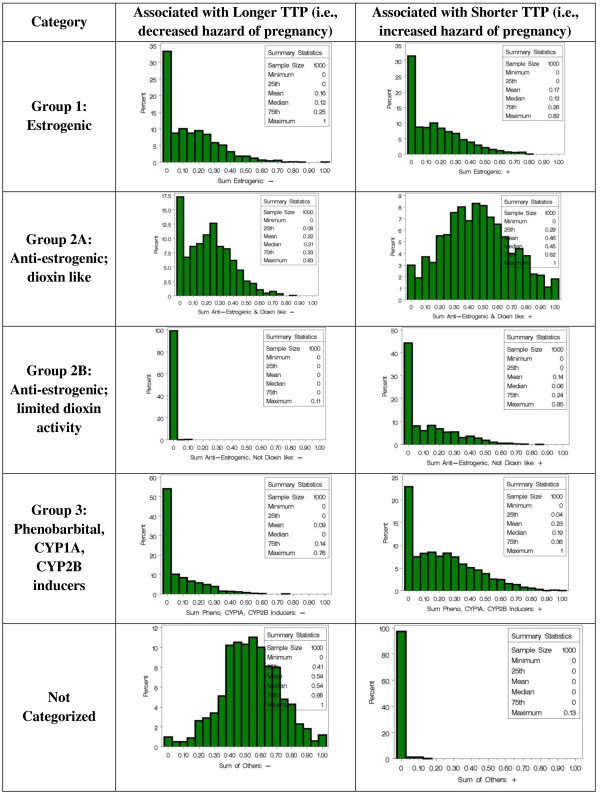
**Bootstrap distributions of the sum of weights within the Wolff et al. classifications of PCBs.** The ‘other’ category includes PCB56 and PCB146.

Parameter estimates were found for the nonlinear Weibull proportional hazards model parameterized with the weights for the 18 PCBs and two covariates (race and breastfed indicators; equation (1)) using the trust region method. A bootstrap analysis was conducted to account for the variation/distribution of the weights. Both positive and negative slope estimates were evaluated using constraints on the coefficient when necessary. Supplementary figures include histograms for the bootstrap distributions for each PCB in a weighted quartile score associated with a positive effect on hazard of pregnancy (Additional file 1: Figure S1) and with a negative effect on the hazard (Additional file 1: Figure S2). These were used to construct histograms for the sum of weights within each functional category (Figure 1). In the analysis associated with longer TTP,

● the average weight from 1000 bootstrap distributions was 16% from estrogenic PCBs;

● the average weight was 22% from anti-estrogenic and dioxin-like PCBs;

● the average weight was 54% from the ‘other’ category; and

● the average weight for the other two groups were less than 10%.

This is in contrast to the weight distributions in the analysis associated with shorter TTP, a more complex scenario:

● the average weight associated with estrogenic PCBs was 17%;

● the average weight associated with anti-estrogenic PCBs that are dioxin-like was 45%;

● the average weight associated with anti-estrogenic PCBs that have limited dioxin activity was 14%; and

● the average weight for the PB-inducers was 23%.

**Figure 2:**
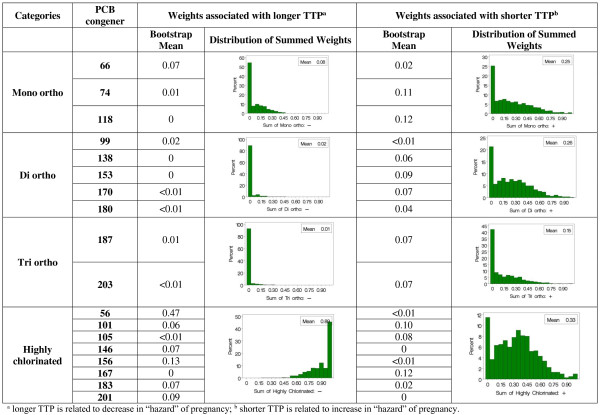
**Average bootstrap estimated weights for PCB congeners related to TTP after adjusting for race (African American vs all other) and whether the daughter was breastfed and grouped according to the degree of chlorine substitution on the ortho position (Kezios et al., **[1]**).**

For the chlorination categorization (Figure 2) in the analysis of longer TTP, the highly chlorinated PCBs were mostly related to longer TTP with average weight of 89%. In contrast, the degree of chlorine substitution was not as discriminating for short TTP with the average weights mostly similar (25%, 26% and 32% for mono, di, and higher) with the exception of lower weights for the tri-ortho group (14%).

A final model was estimated including the two initial covariates, the positive PCB score (using average bootstrap weights, Table 1), the negative PCB score (using average bootstrap weights, Table 1), and the maternal variables of age, BMI, cholesterol and triglycerides. None of the covariates were significant. Both PCB scores had p values less than 0.001; these p values should not be strictly interpreted, as the weighted scores were not *a priori* defined.

**Table 1:** Average bootstrap estimated weights for PCB congeners related to TTP after adjusting for race (African American vs all other) and whether the daughter was breastfed (yes or no)

**Categories**	**PCB congener**	**Weights associated with longer TTP**^**a**^		**Weights associated with shorter TTP**^**b**^	
		**Bootstrap Means**	**Average Bootstrap Sum**	**Bootstrap Means**	**Average Bootstrap Sum**
**Group 1: Estrogenic**	**101**	0.06	16%	0.10	17%
	**187**^**c**^	0.01		0.07	
	**201**	0.09		0	
**Group 2A: Anti-estrogenic (AE), dioxin like**	**66**	0.07	22%	0.02	46%
	**74**	0.01		0.11	
	**105**^**d**^	<0.01		0.08	
	**118**	0		0.12	
	**156**^**c**^	0.13		<0.01	
	**167**	0		0.12	
**Group 2B: AE &not dioxin like**	**138**^**d**^	0	<1%	0.06	14%
	**170**	<0.01		0.07	
**Group 3: Phenobarb, CYP1A, CYP2B inducers**	**99**^**c**^	0.02	9%	<0.01	23%
	**153**	0		0.09	
	**180**	<0.01		0.04	
	**203**	<0.01		0.07	
	**183**^**d**^	0.07		0.02	
**Not categorized**	**56**	0.47	54%	<0.01	0
	**146**	0.07		0	

^a^longer TTP is related to decrease in “hazard” of pregnancy.

^b^shorter TTP is related to increase in “hazard” of pregnancy.

^c^congeners found by Cohn et al. [4] to be associated with longer TTP.

^d^congeners found by Cohn et al. [4] to be associated with shorter TTP.

**Figure 3:**
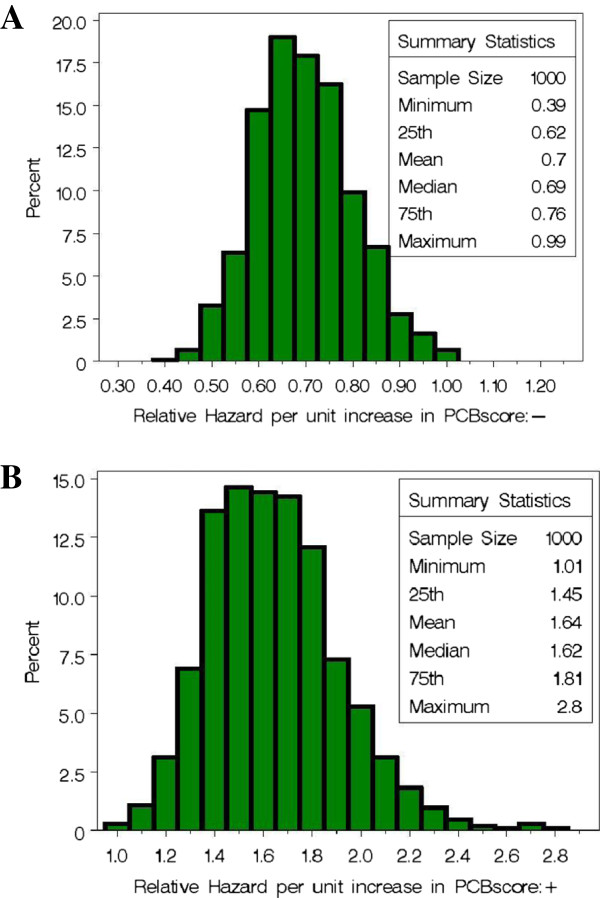
**Bootstrap distribution of the estimated relative hazard of pregnancy based on a unit increase in the PCB scores associated with (A) a decrease in hazard (average score weights are provided in Table**1**, column 3) and (B) an increase in hazard (average score weights are provided in Table**1**, column 5).**

Bootstrap distributions of the relative hazard associated with a positive slope estimate and a negative slope estimate (based on different sets of PCBs) from the final model are provided in Figure 3. On average, the relative hazard of pregnancy associated with longer TTP is 0.70 per unit increase using the weights in Table 1, column 3. On average, the hazard of pregnancy increases 64% per unit increase in the PCB score (i.e., a relative hazard of 1.64) associated with shorter TTP using the weights in Table 1, column 5.

**Figure 4:**
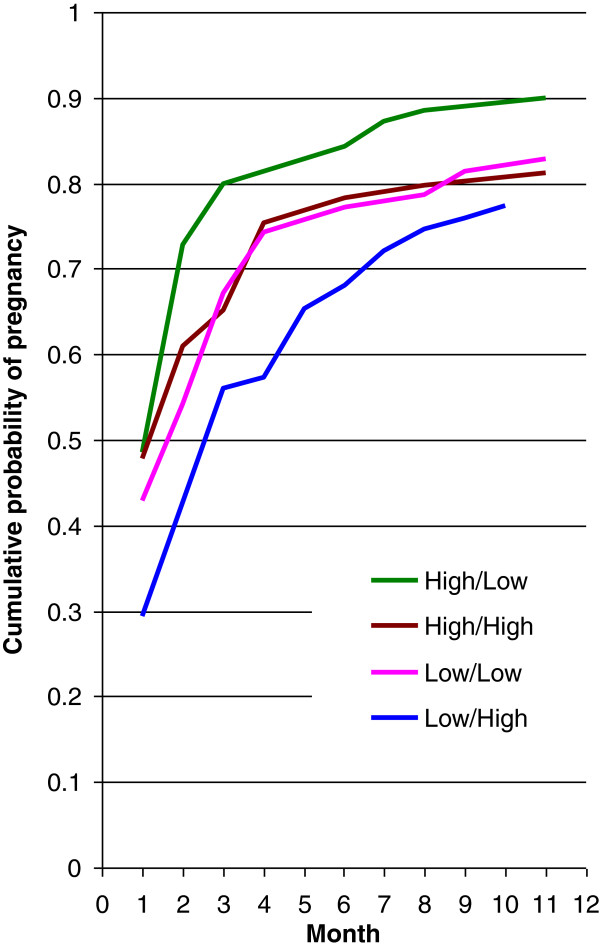
The cumulative probability of pregnancy over cycle months in the four cases defined by the two PCB scores (i.e., PCB+, PCB-) cut at their medians to form the (High, Low), (High, High), (Low, Low) and (Low, High) groupings where PCB + is the score associated with a positive hazard slope (shorter TTP) and PCB- is the score associated with a negative hazard slope (longer TTP).

Finally, the cumulative probability of pregnancy was calculated for the four cases defined by the two scores (PCB + and PCB-) cut at their medians (Figure 4). The combination with the shortest TTP (high concentrations of PCB + and low concentrations of PCB-) has roughly a 10-20% increase in the probability of pregnancy compared to the combination with the longest TTP (low concentrations of PCB + and high concentrations of PCB-). In addition, the plot indicates the cumulative probability of pregnancy increases most rapidly roughly within the first three months.

## Discussion

A previous paper [4] demonstrated that *in utero* exposure to some PCBs may impact human reproduction, either by increasing or decreasing the time to pregnancy. In the studied population, women were exposed to a mix of PCB congeners before birth that predicted longer and shorter TTP and a reduced probability of contraception 30 years later.

The analysis of these same data using an alternative approach provides further insight into the possible link between different categories of PCB functionality and TTP. Using a weighted quartile score approach within a parametric proportional hazards model, empirical estimates of weights per PCB congener were estimated in a weighted quartile score that was positively associated with the hazard of pregnancy (i.e., shorter TTP) and a score that was negatively associated with the hazard of pregnancy (i.e., longer TTP). Generally, different congeners were identified in the two scores. In particular, using a bootstrap analysis to accommodate the variation in the estimated weights, the dominant functionality groups associated with longer TTP were the dioxin-like, anti-estrogenic group (average, 22%) and the ‘uncategorized’ group (PCBs not characterized by Wolff et al.; 54%). In contrast, the uncategorized group was not important in the association with shorter TTP, and the anti-estrogenic groups and the inducers group played an increased role (60% vs 22%; and 23% vs 9%; respectively). Using the second categorization, the highly chlorinated PCBs were mostly associated with longer TTP (89% of the weight), which was not so for short TTP.

Interpretation of the weights from weighted quartile sum regression was instructed through simulation studies conducted by Carrico [11] (detailed below). She demonstrated that clusters of high pairwise correlations (as in the PCBs) have individual weights that tend to be smaller compared to components that are less correlated. Thus, the weights should not be interpreted as indicators of relative importance in the association of interest, rather the correlation pattern should be considered. Here, since the pairwise correlations for PCB 56 were generally low (mean = 0.18, standard deviation =0.12; maximum = 0.39) and the weight was considerably larger than any other weight, we conclude there is an interpretable association with longer TTP.

Carrico [11] conducted extensive simulation studies to characterize WQS regression using environmentally relevant correlation patterns among mixture components. The strength of association between subsets of components and a continuous response variable were varied between correlations of 0.1 and 0.3 using observed correlation patterns from urinary concentrations of environmental chemicals from NHANES data. The method was evaluated in terms of the number of correctly identified bad actors and the number of incorrectly identified components. With a sample size of 250 in 1000 simulated studies where 8 of 11 components were associated with Y with a correlation of 0.3, WQS correctly identified at least 7 of the 8 components 75% of the time with none incorrectly identified more than 95% of the time. When the correlation decreased to 0.1 (a case of poor power), WQS correctly identified 4 or more of the 8 components 80% of the time with none incorrectly identified 35% of the time and in 60% of the cases only selected one component incorrectly. Carrico also conducted simulation studies using LASSO to detect the bad actors and found it tends to identify the correct bad actors but generally identifies incorrect components with high probability.

In our present study we found increased TTP with PCBs thought to be antiestrogenic (PCBs 66, 74, 105, 118, 156 and 167) as well as those not classified in any of the Wolf et al. [9] groupings (PCBs 56, 146). In contrast, shorter TTP was associated with PCBs thought to be anti-estrogenic (PCBs 66,74,105, 118, 156 and 167) and those thought to be PB-inducers (PCBs 99, 153, 180, 203 and 183). Buck Louis et al. [16] in the LIFE study also found reduced fecundability with maternal PCB congeners 118 and 167. They also found reduced fecundability with paternal PCB congeners 138, 167 and 170; for the latter two we found shorter TTP. Because the exact mechanisms by which these associations are likely, it is hard to discern why the results differed for paternal exposure patterns. One possible explanation is that we considered empirically derived mixtures of congeners while Buck Louis evaluated congeners individually.

Recent literature suggests that the developing reproductive system may be influenced by maternal exposures and that such outcomes may differ by sex. Jensen, et al. [17] in a study of Danish twins reported reductions in female fecundity and no relationship with male fecundity related to maternal smoking during pregnancy but not to exposure during childhood. These results support some earlier data ([18,19]) but not other data ([20,21]). Nevertheless, the data support animal studies suggesting that male and female mouse fetuses exposed to polycyclic aromatic hydrocarbons, the major toxic component of cigarette smoke, have reduced fertility, although the precise mechanisms of this association are unknown. More recent data followed a cohort of children exposed to PCBs as well as polychlorinated dibenzo-p-dioxins (PCDD/Fs) in utero and assessed reproductive development at age 8 [22]. Girls exposed to the highest levels of PCBs (either individual congeners or total) had lower genital developmental ages and shorter fundi and uteri lengths and both boys and girls exposed to high levels of total toxic equivalents (TEQ; a combination of PCDD/Fs and specific dioxin like PCBs) had reduced concentrations of estradiol. These results are consistent with previous data that find reduced E2 levels among men exposed to TCDD during infancy [23] and with data that find delayed breast development in girls exposed to PCDD/Fs in utero [24]. Although the precise biological mechanism for these associations is not known, it is thought that these endocrine disrupting chemicals may interfere with the normal action or regulation of reproductive endocrine hormones [25], with estrogen catabolism [26] or with alterations in the aryl hydrocarbon receptor signaling system [27]. Delayed development, particularly in female 8-year olds support our results of reductions in fecundity among women exposed in utero to specific PCBs.

We are aware of no other prior studies of the impact of maternal perinatal serum levels of organochlorine compounds on time to pregnancy in daughters decades later, other than our work in the Child Health and Development Studies cohort [4,28]. Previous work (e.g. [12]) has considered exposures during pregnancy and TTP only in the same (mother’s) generation. Data from the National Collaborative Perinatal Project find inconclusive evidence regarding maternal concurrent exposure to organochlorines and TTP again in the maternal generation [29]. Results to date suggest that developmental exposures, like those that we report here, are more likely to affect time to pregnancy.

When we published our first paper on in maternal PCBs and daughter’s TTP [4], there was active work to determine whether the classification proposed by Wolf and colleagues [9] demonstrated predictive validity for a number of outcomes. By now it appears not to hold up well as we noted in our 2011 TTP paper [4] and in a recent paper on maternal breast cancer [30] and in other papers on infant health outcomes in the CHDS [1]. It is for this very reason that we attempted an alternative approach that did not require a priori classification. In reporting our results, we did consider the Wolff et al. classification in order to provide information in this paper that could be compared to other papers based on this widely used classification. We do not mean to suggest that this classification is better than any other, but we are suggesting that an empirical approach, independent of a priori classification, can be very useful in light of a gap in understanding the biological effects of simultaneous exposure to multiple congeners in different proportions. The chlorination grouping demonstrated the benefit of combining a hypothesized categorization with an empirical approach in the finding that the higher chlorinated PCBs are mostly associated with longer TTP and not shorter TTP.

We were unable to stratify analysis by gravidity, as sample sizes would have been too small for analysis by the methods we used here. However, we previously compared results for daughters who were nulligravidas (N = 54), primigravidas (N = 70) and multigravidas (N = 159) in our prior publication that is the basis for this comparative methods study (Table 3 in [4]). In that comparison we showed, as expected, that TTPs were shorter for parous women, but that overall, PCB associations observed were consistent for all three gravidity groups. Current levels of PCBs were not assessed, but maternal and adult daughter exposures to tobacco (including passive smoking), alcohol and caffeine, daughter’s health history, including sexually transmitted diseases, race, education, income (mother and daughter), daughter’s history of oral contraceptive use and other contraceptive use, marital status, age at menarche, abortion history , age of mother at conception, and age of daughter at pregnancy or attempt were assessed. There was no evidence that these risk factors explained PCB associations [4] and so were not specifically investigated here.

It would have been ideal to measure maternal PCBs at multiple points in gestation in relation to daughter’s TTP 30 years later. However, it was our objective to conserve valuable maternal prenatal serum samples for other research questions that require precisely timed sampling, such as steroid hormones and thyroid hormones. We thus chose to use maternal postpartum samples as a proxy for daughter’s *in utero* exposure to PCBs. While it is possible that some PCB congeners are more subject to change during pregnancy [31], the postpartum blood samples in our study were drawn within 1–3 days of delivery, minimizing this source of misclassification. Based on rates of daily change in PCB levels reported by Bloom and colleagues [31], variability due to changes in PCB levels over three days after delivery would be small and considerably less than the substantial inter-individual variability of maternal PCB levels we observed in this sample [4]. For example, based on mean daily rate of change estimates presented in Bloom et al., ([31], Table 4), these three days past delivery in our study would likely result in no or in extremely small differences between *in utero* PCB levels and measured PCB levels in postpartum samples collected so soon after delivery. Consistent with this argument, Longnecker et. al suggested that using postpartum maternal samples does not introduce serious misclassification in ranking daughters according to their *in utero* PCB exposures, since inter-individual variation in PCB levels greatly exceeds intra-individual variation due to timing of sampling [32].

One potential source of bias in this and our previous study is the retrospective assessment of time to pregnancy. As is typical, we chose a menstrual cycle as the time unit as each cycle offers a single ovulatory opportunity for pregnancy to occur [33]. Numerous problems with this and other (i.e. prospective assessments) are described in the literature ([33-38]). As described in the methods, we asked each woman to report on her most recent pregnancy to avoid recall bias; nevertheless, as reported by Cooney et al. [38] and commented upon by Joffe [39], retrospective recall of TTP may result in misclassified true values, with the degree of measurement error proportional to gravidity, the time since pregnancy and duration of TTP. In the study of Cooney, et al. [38], where women who participated in a prospective study of TTP reported the same TTP retrospectively 10 years later, the distribution of reporting errors appears skewed to the left; however the sample size was small. In our study, where women were still reproductive age at interview (ages 27–31 years), the median time from the reported pregnancy to questionnaire was 3 years, with 25% reporting within 1 year and 75% reporting within 5 years of their most recent pregnancy or pregnancy risk period, suggesting that the bias may be smaller. As also indicated above in the methods, we included women who omitted birth control just once, women who became pregnant during an interval between birth control methods or after stopping birth control, women who became pregnant when breast feeding, women who never used birth control, women who were attempting pregnancy after a prior pregnancy, and women who never became pregnant, but had been at risk for pregnancy for a known interval, whether or not they desired to become pregnant. Many of these inclusions are an attempt to minimize other biases in the retrospective ascertainment of TTP as discussed by Joffe et al. [35], Key et al. [37] and Scheike and Keiding [36].

There are several studies which evaluate the associations between exposure to PCBs and other chlorinated compounds with regard to pregnancy. With one exception, previous studies base their exposure assessment on either fish consumption (paternal - [40]; maternal - [41]) or serum measures ([16,29,41-44]) proximal to the study. The notable exception is the analysis of exposure to contaminated cooking oil in the Yucheng cohort [45]; analyses here performed for women exposed during childhood and adolescence. Thus, our study ([4] and reported here) is unique in that it focuses on developmental exposure (i.e. during the in utero period) and fecundability outcomes in the female adult offspring. In Cohn et al. [4], we found longer TTP associated with prenatal exposure to PCBs 187, 156 and 99 and shorter TTP associated with prenatal exposure to PCBs 105, 138, and 183.

Women exposed to PCB/dibenzofuran/dibenzodioxins contaminated cooking oil [45] experienced prolonged TTP and reduced fertility, reported 24 years after the accident. Although TTP was queried for all pregnancies, only the first pregnancy was used in the analysis to minimize dependencies on subsequent TTP. TTP was defined as the duration between discontinuing contraception and the beginning of the last menstrual period prior to conception. TTP in exposed women was reduced by approximately 1 month compared to controls not exposed. We note that the Yucheng cohort has documented high exposure levels to this mixture of contaminants.

The New York State Angler’s Cohort is a prospective cohort study, which was initiated in 1991 as a result of concern regarding the role of environmental contaminants and reproductive outcomes. Prospective analyses using a subset of 102 women suggest that the weakly antiestrogenic PCB congener, PCB 205 and weakly estrogenic congener, PCB 206, as well as the antibacterial hexaclorobenzene were associated with increased TTP among women who became pregnant in the first three cycles following cessation of contraception [42]. Further analyses found reduced fecundity for an a priori defined group of estrogenic PCB congeners (4_10, 5_8, 15_17, 18, 44, 47, 48, 52, 70, 99, 101, 136, 153 and 188) and a group of antiestrogenic PCB congeners (77_110, 105, 114, 126, 171_156, and 169), although the confidence limits surrounding the point estimates were wide and include the null [43].

An analysis of Swedish fishermen’s wives and sisters [44] found decreased TTP among those with PCB153 concentrations in the middle and high tertiles, compared to the lowest tertile, leading the investigators to posit that concentrations may not have been high enough to find an adverse association. All data collected in this study were retrospective and TTP was based on the first planned pregnancy. Moreover, PCB153 concentrations at the time of the first planned pregnancy were estimated based on a toxicokinetic model relating serum concentrations measured at the time of study, number of children, and breast feeding history. PCB153 is included in the group of estrogenic congeners but may not be the best for estimating exposure.

Data from the National Collaborative Perinatal Project [29] found a small association between exposure to the sum of 11 PCB congeners and TTP, such that women with concentrations in the highest quartile had longer TTP. TTP was assessed by questionnaire to the group of pregnant women. More recently, results from the LIFE study [16] reduced fecundability was associated with concentrations of individual PCB congeners (PCBs 118, 167, 209). Notably, the LIFE study prospectively recruited women attempting pregnancy following discontinuation of contraception. A final study considered the associations between PCBs and TTP among a subcohort of 332 pregnant women in the PELAGIE cohort [41]. TTP was assessed via questionnaire. Reduced fecundability was found for exposure to the 10 PCB congeners (138, 153, 180, 118, 170, 187, 194, 183, and 203) and for the sum of the congeners. Many of the estimated associations were reduced with control for shellfish consumption.

In sum, there is some evidence of reduced fecundability and increased time to pregnancy in previous studies which consider both exposure and outcomes measured in adult women. Associations, however, are modest and conflicting in regard to which PCB congener or groups of congeners are important. Our study of *in utero* PCB exposure in relation to daughter’s fecundability is the only study to date where exposure was measured at this potentially critical window of susceptibility.

## Conclusions

Results of the present study suggest that methods based on *a priori* classification of compounds could miss the contribution of compounds that have not been or cannot be classified. We suggest that empirical approaches, such as the one we report here, could supplement approaches based on expected biological activity or structure of mixture components. These supplemental empirical methods can generate new hypotheses and may stimulate the interest of toxicologists and basic scientists to investigate and explain the patterns reported. We suggest that the strategy of moving from observation in a human cohort to experimental science in the laboratory could accelerate discovery of new or different mechanisms.

## Consent

Participants gave informed consent for a study on how early life factors influence reproduction with publications in aggregate form.

## Abbreviations

CHDS: Child Health and Development Studies; E2: Estradiol; NHANES: National Health and Nutrition Examination Survey; PCB: Polychlorinated biphenyls; PCDD/F: Polychlorinated dibenzo-p-dioxins; PELAGIE: A French birth cohort described in Chevrier et al. [41]; Proc: Procedure; TCDD: 2,3,7,8-Tetrachlorodibenzodioxin; TEQ: Toxic equivalent quotients; TTP: Time to pregnancy; WQS: Weighted quartile sum.

## Competing interests

The authors declare that they have no competing interests.

## Authors’ contributions

CG contributed to the statistical methods development, performed the statistical analysis, and wrote the first draft of the paper. CC contributed to the statistical methods development. PF-L participated in the design and coordination of the study and helped draft the manuscript. NK assisted in data management. PC and BC participated in the design and coordination of the study and helped draft the manuscript. All authors read and approved the final manuscript.

## Supplementary Material

Additional file 1: Figure S1 Histograms for bootstrap distributions of weights for the PCB congeners in the weighted quartile score with a positive slope parameter in a Weibull proportional hazards model adjusted for race (African American vs all other) and whether the daughter was breast fed (yes or no). **Figure S2**. Histograms for bootstrap distributions of weights for the PCB congeners in the weighted quartile score with a negative slope parameter in a Weibull proportional hazards model adjusted for race (African American vs all other) and whether the daughter was breast fed (yes or no). **Table S1**. Correlation estimates for the PCB congeners based on maternal serum concentrations (N = 289). Click here for file
