# Supplementary material for: A Cohort study evaluation of maternal PCB exposure related to time to pregnancy in daughters
Source: Environ Health. 2013 Aug 20;12:66. doi: 10.1186/1476-069X-12-66 (PMC3766643; doi:10.1186/1476-069X-12-66)
Supplement: Additional file 1: Figure S1 — Histograms for bootstrap distributions of weights for the PCB congeners in the weighted quartile score with a positive slope parameter in a Weibull proportional hazards model adjusted for race (African American vs all other) and whether the daughter was breast fed (yes or no). Figure S2. Histograms for bootstrap distributions of weights for the PCB congeners in the weighted quartile score with a negative slope parameter in a Weibull proportional hazards model adjusted for race (African American vs all other) and whether the daughter was breast fed (yes or no). Table S1. Correlation estimates for the PCB congeners based on maternal serum concentrations (N = 289). [file 1476-069X-12-66-S1.pdf]

Additional file Figures and Table

Figure S1: Histograms for bootstrap distributions of weights for the PCB congeners in the weighted quartile score with a positive slope parameter in a Weibull proportional hazards model adjusted for race (African American vs all other) and whether the daughter was breast fed (yes or no).

Estrogenic and persistent

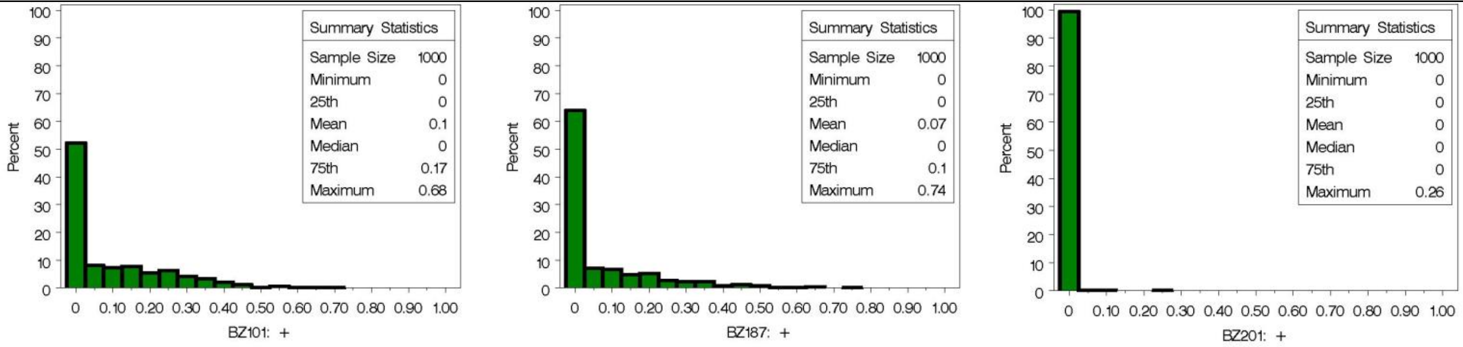

Anti-estrogenic, immunotoxic, dioxin like (non-ortho, mono-ortho)

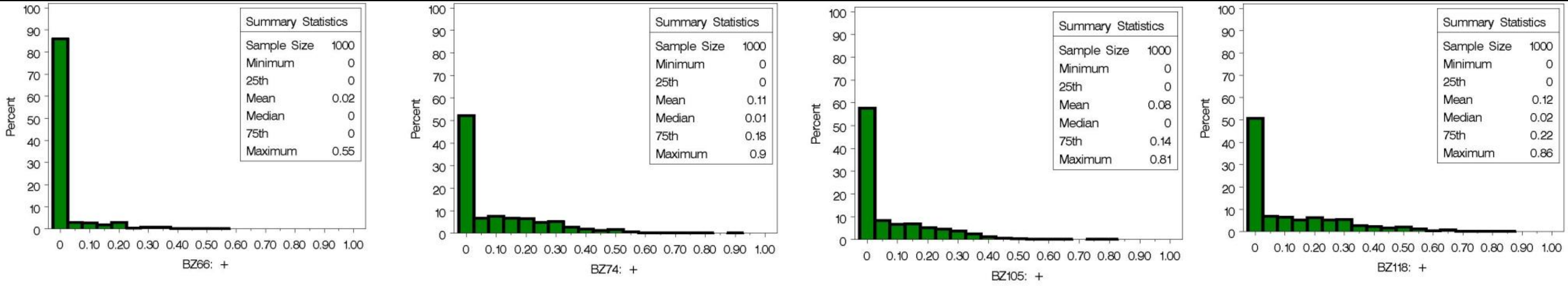

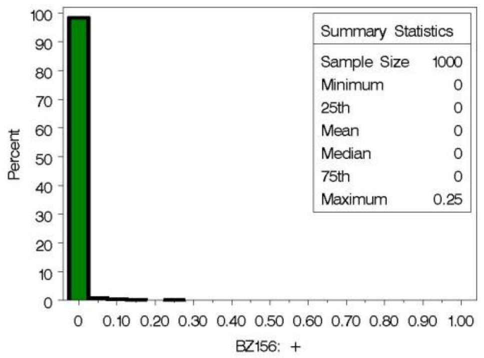

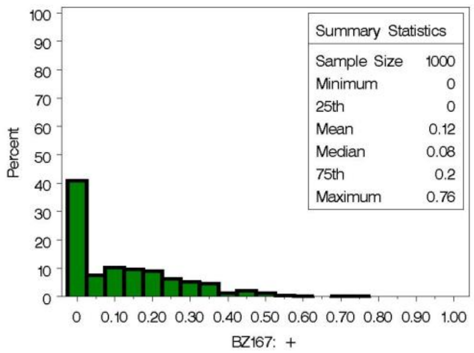

Anti-estrogenic, immunotoxic, limited dioxin-like activity (di-ortho)

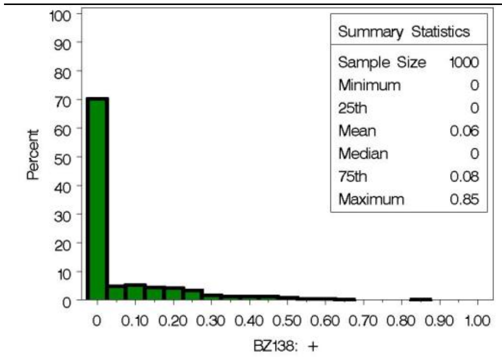

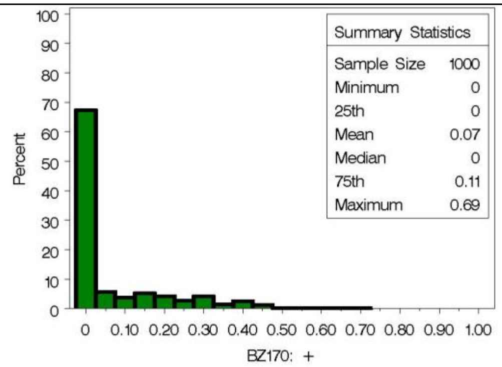

Phenobarbital, CYP1A, CYP2B inducers

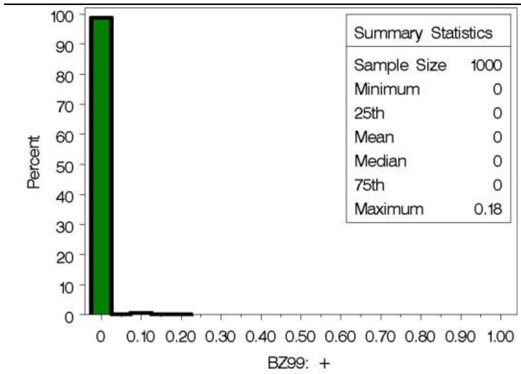

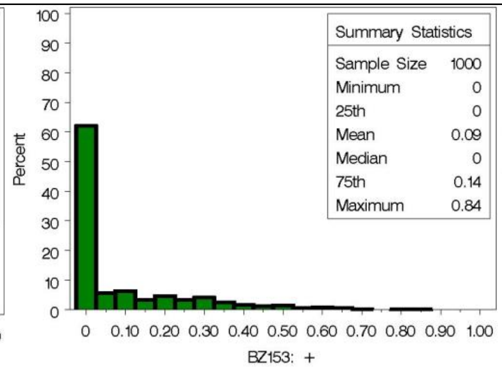

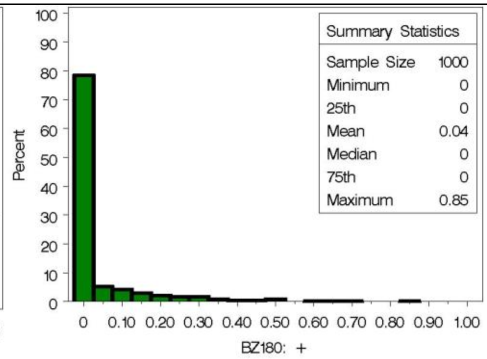

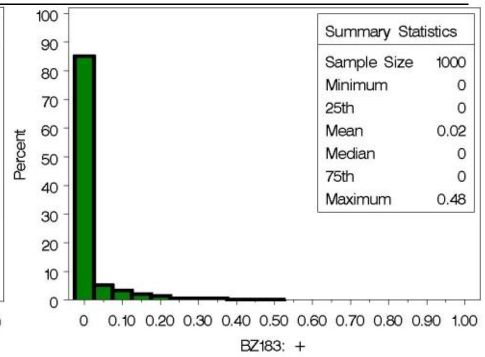

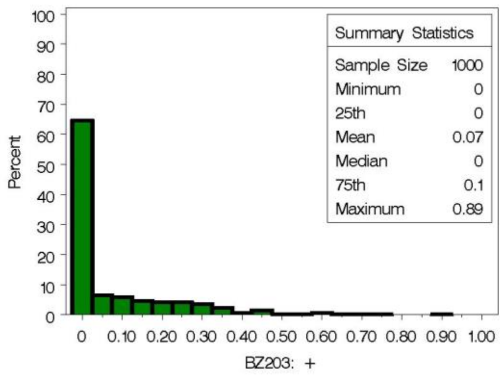

Not categorized by Wolff et al

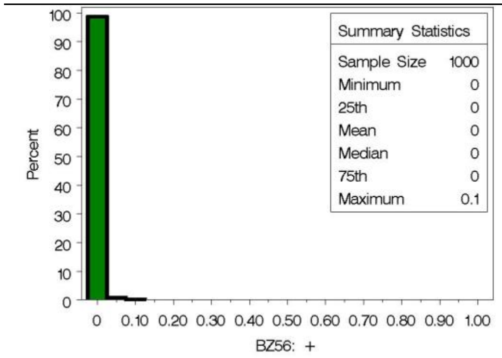

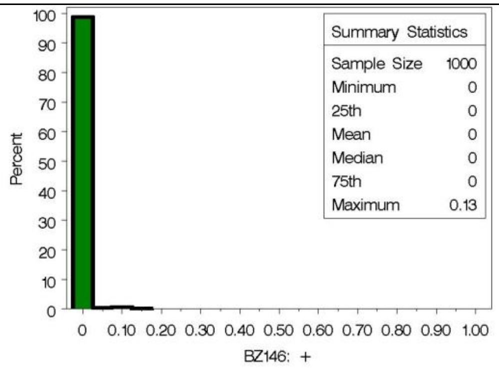

Figure S2: Histograms for bootstrap distributions of weights for the PCB congeners in the weighted quartile score with a negative slope parameter in a Weibull proportional hazards model adjusted for race (African American vs all other) and whether the daughter was breast fed (yes or no).

Estrogenic and persistent

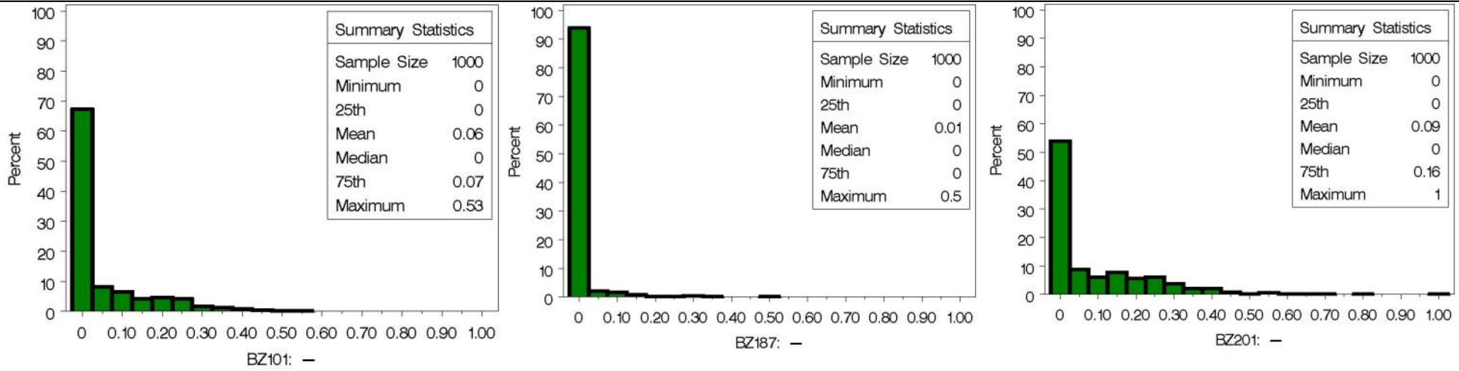

Anti-estrogenic, immunotoxic, dioxin like (non-ortho, mono-ortho)

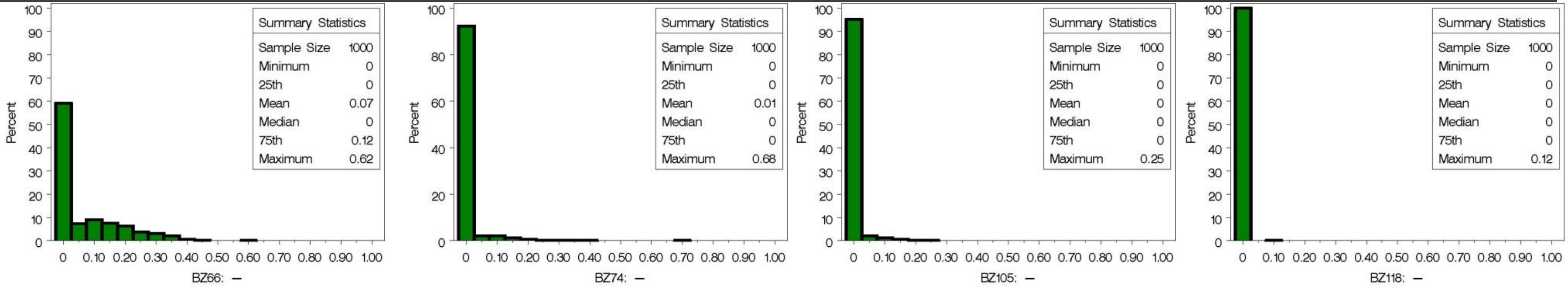

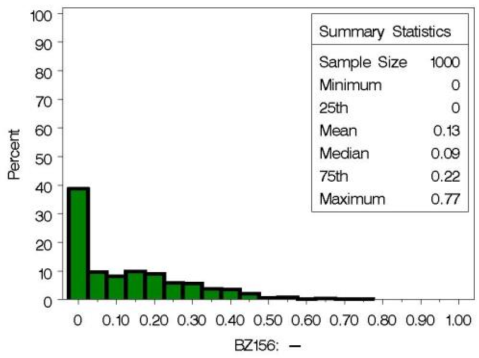

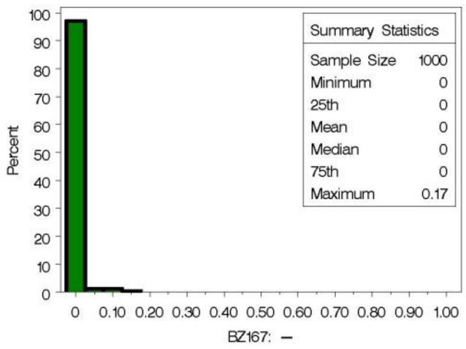

Anti-estrogenic, immunotoxic, limited dioxin-like activity (di-ortho)

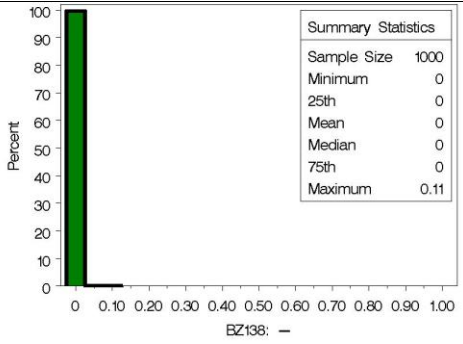

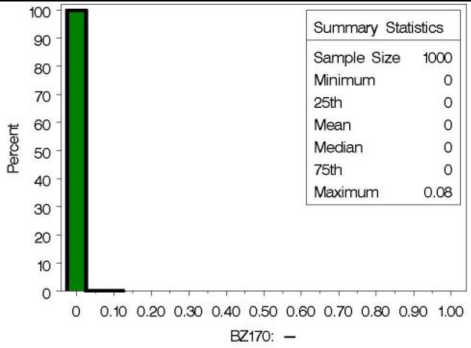

Phenobarbital, CYP1A, CYP2B inducers

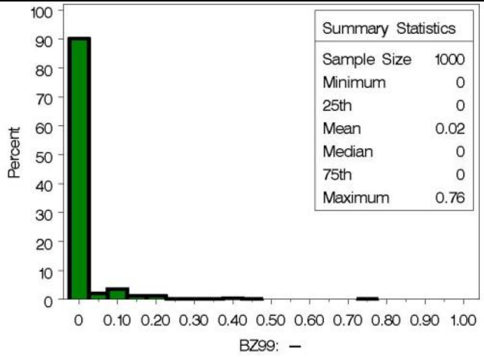

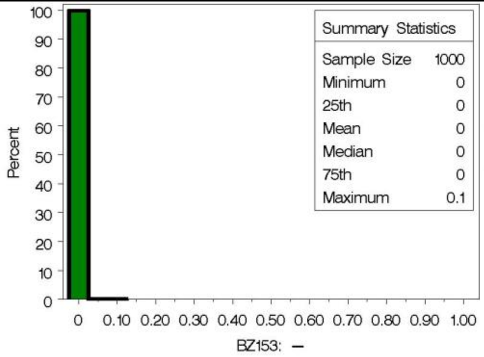

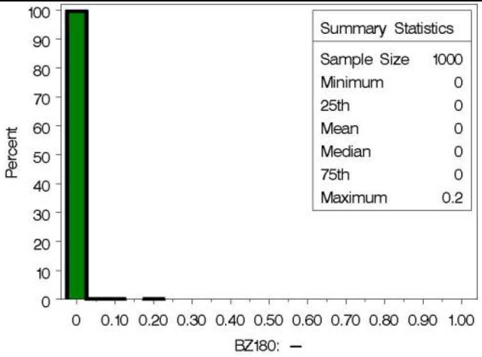

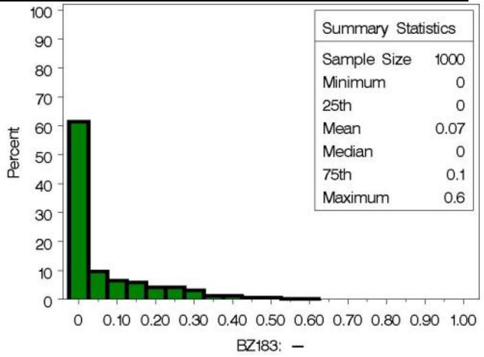

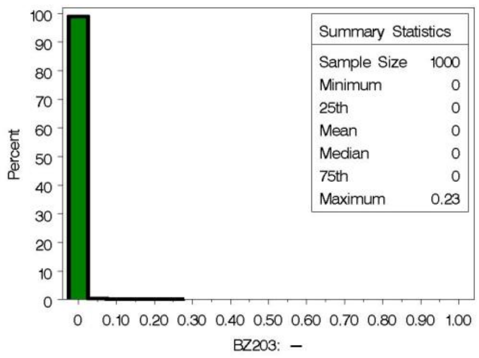

Not categorized by Wolff et al

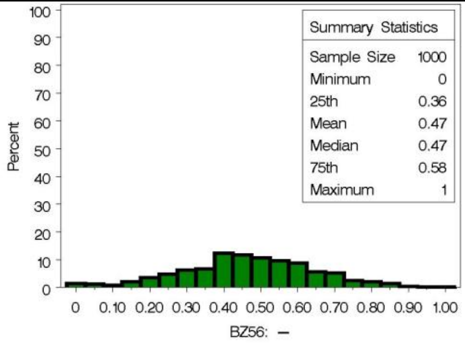

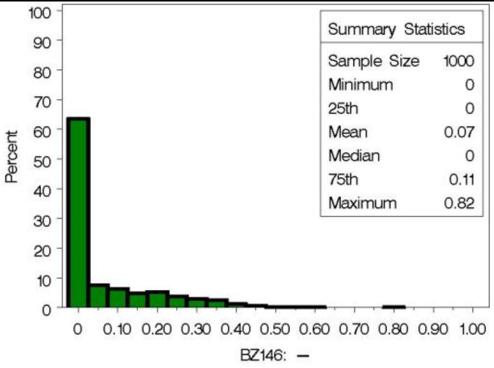

Table S1: Correlation estimates for the PCB congeners based on maternal serum concentrations (N=289)

Categories	PCB	101	187	201	66	74	105	118	156	167	138	170	99	153	180	203	183	56	146
**Group 1**	**101**	1	0.81	0.45	0.44	0.16	0.32	0.45	0.51	0.49	0.72	0.58	0.21	0.73	0.71	0.64	0.77	0.16	0.66
	**187**		1	0.72	0.14	0.09	0.39	0.50	0.60	0.52	0.88	0.80	0.21	0.90	0.94	0.81	0.87	0.23	0.87
	**201**			1	-.14	0.13	0.43	0.42	0.55	0.51	0.64	0.66	0.28	0.69	0.76	0.81	0.66	0.39	0.73
**Group 2A**	**66**				1	0.14	0.09	0.28	0.15	0.15	0.22	0.14	0.03	0.20	0.15	0.10	0.27	-.11	-.03
	**74**					1	0.38	0.45	0.20	0.15	0.20	0.15	0.50	0.21	0.14	0.18	0.10	-.01	0.15
	**105**						1	0.73	0.34	0.37	0.44	0.42	0.57	0.50	0.45	0.43	0.44	0.15	0.45
	**118**							1	0.46	0.34	0.69	0.57	0.74	0.67	0.56	0.53	0.48	0.07	0.53
	**156**								1	0.45	0.74	0.61	0.37	0.76	0.66	0.51	0.57	0.20	0.67
	**167**									1	0.49	0.61	0.31	0.54	0.52	0.53	0.61	0.36	0.59
**Group 2B**	**138**										1	0.86	0.43	0.97	0.91	0.77	0.77	0.19	0.87
	**170**											1	0.36	0.86	0.88	0.80	0.75	0.22	0.84
**Group 3**	**99**												1	0.38	0.25	0.28	0.25	0.15	0.34
	**153**													1	0.95	0.80	0.80	0.19	0.90
	**180**														1	0.87	0.82	0.19	0.89
	**203**															1	0.74	0.23	0.78
	**183**																1	0.27	0.76
**Not categorized**	**56**																	1	0.28
	**146**																		1
